# Development of High-Throughput Method for Measurement of Vascular Nitric Oxide Generation in Microplate Reader

**DOI:** 10.3390/molecules22010127

**Published:** 2017-01-13

**Authors:** Soad S. Abd El-Hay, Christa L. Colyer

**Affiliations:** 1 Faculty of Pharmacy, Pharmaceutical Chemistry Department, King Abdulaziz University, Jeddah 21589, Saudi Arabia; 2 Faculty of Pharmacy, Department of Analytical Chemistry, Zagazig University, Zagazig 44519, Egypt; 3 Department of Chemistry, Wake Forest University, Winston-Salem, NC 27109, USA; colyercl@wfu.edu

**Keywords:** nitric oxide, DAF-FM, SIN-1, fluorescence, microplate reader

## Abstract

Background: Despite the importance of nitric oxide (NO) in vascular physiology and pathology, a high-throughput method for the quantification of its vascular generation is lacking. Objective: By using the fluorescent probe 4-amino-5-methylamino-2′,7′-difluorofluorescein (DAF-FM), we have optimized a simple method for the determination of the generation of endothelial nitric oxide in a microplate format. Methods: A nitric oxide donor was used (3-morpholinosydnonimine hydrochloride, SIN-1). Different factors affecting the method were studied, such as the effects of dye concentration, different buffers, time of reaction, gain, and number of flashes. Results: Beer’s law was linear over a nanomolar range (1–10 nM) of SIN-1 with wavelengths of maximum excitation and emission at 495 and 525 nm; the limit of detection reached 0.897 nM. Under the optimized conditions, the generation of rat aortic endothelial NO was measured by incubating DAF-FM with serial concentrations (10–1000 µM) of acetylcholine (ACh) for 3 min. To confirm specificity, *N^ω^*-Nitro-l-arginine methyl ester (l-NAME)—the standard inhibitor of endothelial NO synthase—was found to inhibit the ACh-stimulated generation of NO. In addition, vessels pre-exposed for 1 h to 400 µM of the endothelial damaging agent methyl glyoxal showed inhibited NO generation when compared to the control stimulated by ACh. Conclusions: The capability of the method to measure micro-volume samples makes it convenient for the simultaneous handling of a very large number of samples. Additionally, it allows samples to be run simultaneously with their replicates to ensure identical experimental conditions, thus minimizing the effect of biological variability.

## 1. Introduction

Many factors released from the endothelium determine vascular homeostasis, which is the key mechanism of the atherosclerotic process [1]. Nitric oxide is the most important factor released from the endothelium, and controls vascular homeostasis. NO is synthesized in endothelial cells, and activates guanylate cyclases, leading to vasodilation; it also maintains vascular wall homeostasis by inhibiting inflammation [2]. The vasodilation response is undermined by an imbalance in vasodilating and vasoconstricting substances, which causes endothelial dysfunction. So, the measurement of endothelial nitric oxide is of tremendous interest for the evaluation of endothelial function [1].

NO measurement is also essential for researchers who are studying many cellular physiological or pathological processes, especially those related to the cardiovascular system. A literature survey reveals that NO has been determined by chemiluminescence [3], spectrophotometry [4,5,6], electrochemistry [7,8], HPLC [9,10,11], and fluorimetry [12,13,14]. However, these previously published methods can suffer from low sensitivity, long analysis times, or the need for complicated instrumentation; therefore, there remains a need for the development of an improved method.

**Figure 1:**
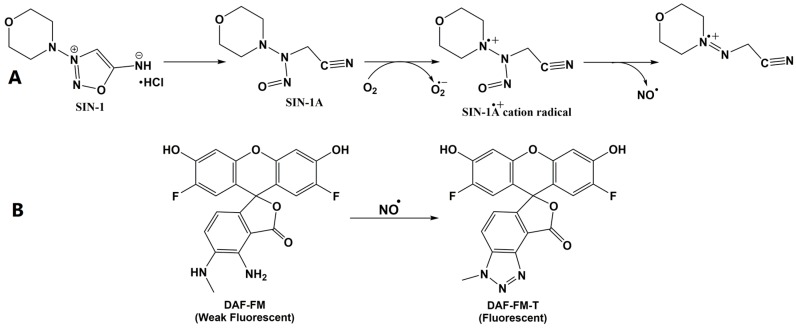
Schematic representation of (**A**) the suggested reaction liberating nitric oxide (NO) from 3-morpholinosydnonimine hydrochloride (SIN-1) and (**B**) the reaction of 4-amino-5-methylamino-2′,7′-difluorofluorescein (DAF-FM) with NO generated from SIN-1.

The fluorescent probe 4-amino-5-methylamino-2′,7′-difluorofluorescein (DAF-FM) is a very sensitive reagent used for the determination of NO. The fluorescent chemical transformation is based on the reactivity of NO with the aromatic vicinal diamines of DAF-FM in the presence of dioxygen, which yields the highly green-fluorescent triazole form (DAF-FM-T); it offers the advantages of sensitivity and specificity, and is a simple protocol for the direct detection of NO [15]. DAF-FM-T shows stable and intense fluorescence over a wide range of pH values [16]. Thus, NO liberated from 3-morpholinosydnonimine hydrochloride (SIN-1) can react with DAF-FM to produce an intensely fluorescent triazole derivative (with detection limit <1 nM), as illustrated in Figure 1.

Previous methods for the detection of NO used the cell-permeable derivative DAF-FM diacetate instead of DAF-FM in cells [17], not tissue as in the current study. A method for the kinetic analysis of DAF-FM activation by NO has been proposed, but it did not include a means of measuring NO in tissue [18]. Other methods require sophisticated instrumentation like confocal laser scanning microscopy to detect NO in plants [19]. In the meantime, there is a continuous demand—especially in drug discovery—for high-throughput analyses, which can perform 96 analyses simultaneously when using the 96-well plate. We have succeeded in determining the generation of vascular endothelial NO using a high-throughput microplate reader, with the potential of applying it to other systems.

## 2. Results and Discussion

### 2.1. Effect of Dye Concentration

**Figure 2:**
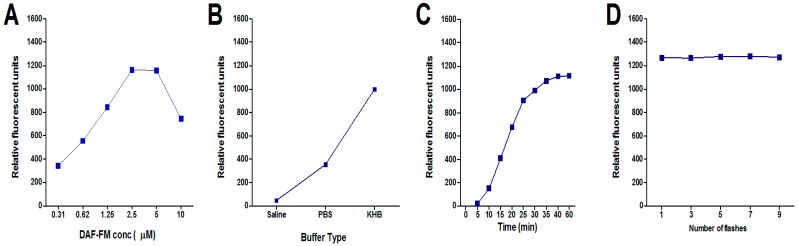
Effect of (**A**) different DAF-FM concentrations; (**B**) different buffer types; (**C**) incubation times; and (**D**) a different number of flashes on the fluorescence intensity of the reaction product of NO generated from 1 µM NO donor (SIN-1). Values shown represent the mean ± standard error of the mean (SEM) for three independent replicated experiments.

The measured fluorescence emission intensity of the sample solution prepared from SIN-1 and DAF-FM increased as the dye concentration increased, until it reached a maximum at a dye concentration of 2.5 µM and then decreased as concentration increased, as demonstrated in Figure 2A. The relative fluorescence intensity was measured at 525 nm with excitation at 495 nm after subtracting blank values.

The decrease in fluorescence could possibly be explained by the inner filter effect, which results in the diminution of the overall fluorescence emission at the higher dye concentration due to reabsorption of emitted light by chromophores in the detector pathway. Additionally, at elevated concentrations of dye, it is possible that dye aggregation is occurring, which would be a competing equilibrium with the dye–NO binding, and so would result in diminished fluorescence emission.

### 2.2. Effect of Buffer

The fluorescence intensity resulting from the interaction between DAF-FM and NO released by SIN-1 gave highest fluorescence when reacted with DAF-FM in Krebs–Henseleit buffer (KHB) than with phosphate buffered saline (PBS) or saline (Figure 2B). This may be because in liberating NO, SIN-1 changes the pH of the medium. The pH affects the reaction between the liberated NO and the DAF-FM. Therefore, using a strong buffer system like KHB improved the obtained fluorescence in the case of SIN-1. Decomposition results not only in the liberation of NO, but also superoxide, which rapidly reacts with NO to form peroxynitrite. However, it seems complex in solutions containing phosphate buffer [20]. It was found that when incubating SIN-1 with deoxyribose in phosphate buffered saline, the quantity of malondialdehyde formed from the oxidation of deoxyribose by peroxynitrite was only 3% of the concentration of SIN-1 calculated to have decomposed [21]. The known vasodilatory effect of SIN-1 confirms that NO is the major molecule generated from its decomposition, while peroxynitrite works against vasodilation [22,23].

### 2.3. Effect of Reaction Time and Fluorescence Stability

The reaction between DAF-FM and SIN-1 took 60 min to complete (Figure 2C). The fluorescence of the complex that formed from the reaction between DAF-FM and the NO was stable even when exposed to repeated stimulation, where it can be seen that the increase in the number of flashes did not affect the measured fluorescence. The stability of fluorescence with increasing number of flashes is critical, as the fluorescence intensity could be quenched by time (Figure 2D).

Fluorescein is widely used as a fluorophore in biology because of its high fluorescence quantum yield in water and its convenient wavelengths for biological measurement, but fluoresceinamine (5-aminofluorescein) was reported to show quenched fluorescence because of the electron-donating group attached to the phthalic ring of fluorescein [24]. However, when the electron-donating group is converted to a less electron-donating group, the fluorescence recovers, and this is the basis of diaminofluorescein (DAF). The reaction with NO via the formation of the triazole ring reduces the electron donating capability of the functional groups attached to fluorescein, leading to an NO concentration-dependent enhancement of fluorescence [15].

### 2.4. Method Validation

Linear relationships between the measured fluorescence and NO donor were obtained under the optimal experimental conditions at the physiological concentrations (1–10 nM) of NO released from the endothelium. The fluorescence quantum efficiencies are known to be increased by a factor of 100 or more after the transformation of DAFs by NO [15]. There was a strong direct correlation between the NO released by SIN-1 and the fluorescence obtained, as indicated by the high coefficient of determination value (r^2^ = 0.976); this is statistically significant (*p* < 0.001), and is supported by low limit of detection (LOD) and limit of quantification (LOQ) values. Good precision was indicated by relative standard deviation (RSD%) values lower than 3% and 4% for intraday and interday precision, respectively. Statistical parameters and linearity results are summarized in Table 1.

### 2.5. Application of the Method in Studying Endothelial NO Generation

**Figure 3:**
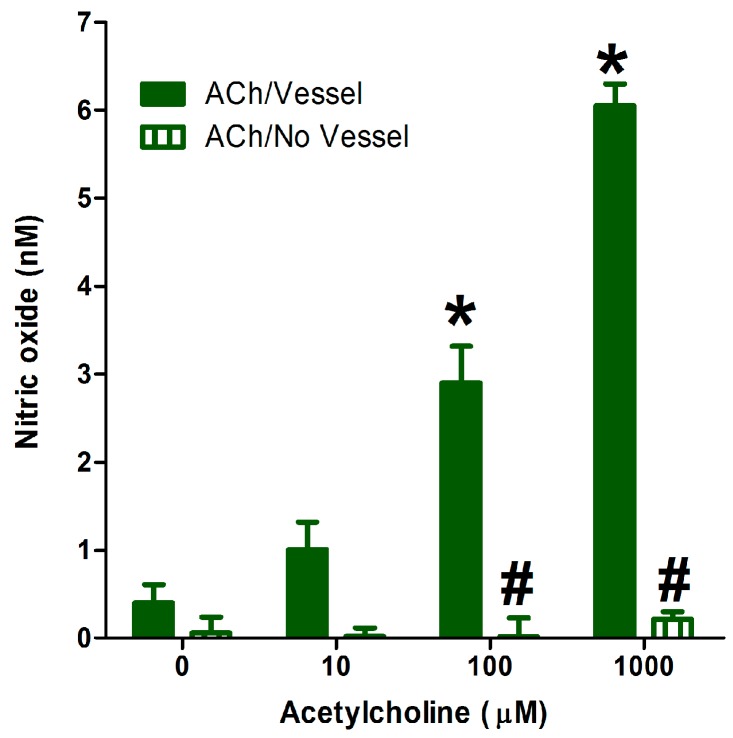
Effect of adding cumulative concentrations of acetylcholine (ACh) in the presence or absence of isolated aortae on nitric oxide generation. Values shown represent the mean ± standard error of the mean (SEM) for four independent replicate experiments. * Significantly different from NO generation before ACh addition at *p* < 0.05. # Significantly different from NO generation at the corresponding ACh concentration at *p* < 0.05 by two-way ANOVA followed by Bonferroni post hoc test.

The addition of cumulative concentrations (1–1000 µM) of acetylcholine (ACh)—the standard endothelial NO-generating molecule—to microplate black wells containing 3 mm isolated aorta in KHB/DAF-FM (2.5 µM) led to a dose-dependent generation of NO. However, the addition of the same concentrations of ACh in KHB/DAF-FM without the aortic vessels did not change DAF-FM fluorescence (Figure 3). This confirms that ACh alone did not interact with the dye to produce an enhanced fluorescence emission. Rather, the ACh stimulates NO release from the aortic vessel, which was seen as an increase in fluorescence in our optimized assay.

**Figure 4:**
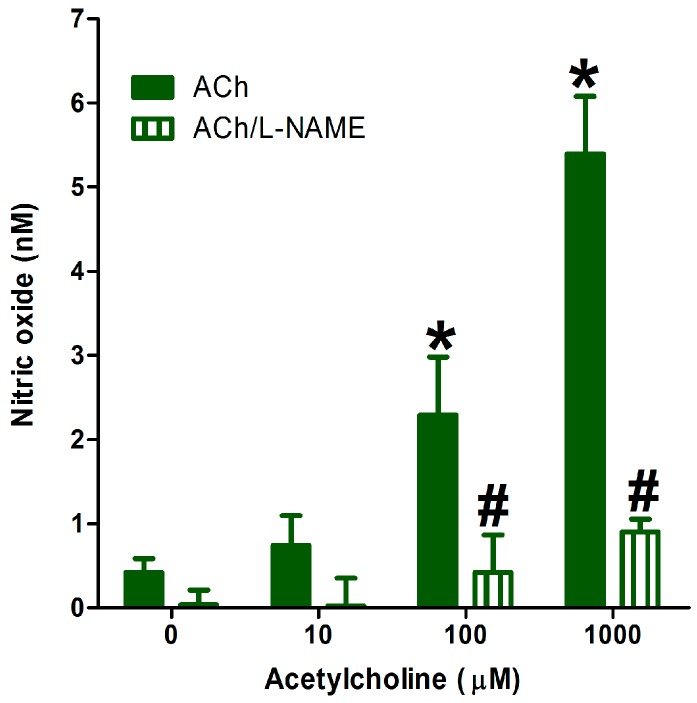
Effect of the addition of cumulative concentrations of ACh on NO generation from isolated aortae in absence or presence of *N^ω^*-Nitro-l-arginine methyl ester (l-NAME, 300 µM). Values shown represent the mean ± SEM for four independent replicate experiments. * Significantly different from NO generation before ACh addition at *p* < 0.05. # Significantly different from NO generation at the corresponding ACh concentration at *p* < 0.05 by two-way ANOVA followed by Bonferroni post hoc test.

The presence of 300 µM *N^ω^*-Nitro-l-arginine methyl ester (l-NAME)—the standard inhibitor of endothelial NO generation—significantly inhibited the ACh-stimulated generation of NO, as Figure 4 shows. This confirms the specificity of the current method in determining endothelial NO generation in the presence of stimulants and inhibitors.

**Figure 5:**
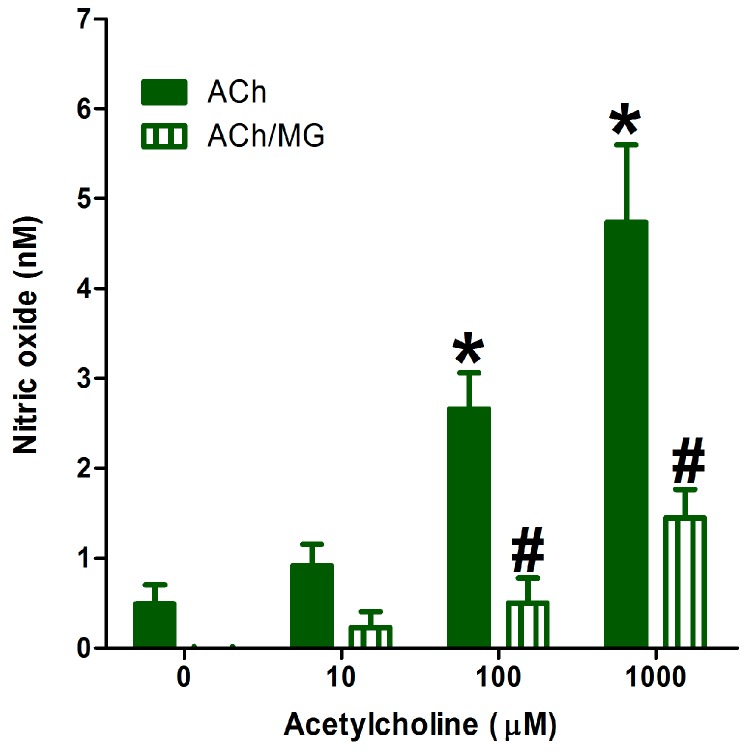
Effect of the addition of cumulative concentrations of ACh on nitric oxide generation from isolated normal aortae or aortae preincubated for 1 h with 400 µM methyl glyoxal (MG). Values shown represent the mean ± SEM for four independent replicate experiments. * Significantly different from NO generation before ACh addition at *p* < 0.05. # Significantly different from NO generation at the corresponding ACh concentration at *p* < 0.05 by two-way ANOVA followed by Bonferroni post hoc test.

Pre-exposing the isolated aortae to 400 µM methyl glyoxal (MG) for 1 h resulted in advanced glycation product, which significantly decreased the endothelial NO generation measured by the current method (Figure 5). This makes the current method useful for studying the effect of different agents or disease conditions on endothelial NO generation. The short analysis time (10 min per sample) required by this new method and its simplicity makes it an attractive high-throughput method for measuring this biologically important molecule.

## 3. Experimental Section

### 3.1. Apparatus

The fluorescence emission intensity was measured in Costar^®^ 96-well black microplate (Fisher Scientific, Pittsburgh, PA, USA) using a monochromator SpectraMax^®^ M3 plate reader (Molecular Devices, Sunnyvale, CA, USA. Fluorescence emission was measured at 525 nm after excitation at 495 nm using a 515 nm emission cutoff and a 200 V gain. The number of flashes was set at two per read, as described in the Results and Discussion section. Solution pH was measured using a Jenway^®^ 3510 Bench pH meter (Fisher Scientific Ltd., Leicestershire, UK).

### 3.2. Reagents

All chemical reagents were of analytical grade, obtained from various commercial suppliers, and used without further purification unless otherwise indicated. DAF-FM, SIN-1, PBS, dimethylsulfoxide (DMSO, molecular grade), *N^ω^*-Nitro-l-arginine methyl ester (l-NAME), methyl glyoxal (MG), and acetylcholine (ACh) were purchased from Sigma-Aldrich Sigma-Aldrich, St. Louis, MO, USA). Pluronic F-127^®^ (20%) was purchased from Molecular Probes (Paisley, UK). Fresh grade I ultrapure deionized water obtained from a Milli-Q^®^ Integral Water Purification System (EMD Millipore, Billerica, MA, USA) was used throughout the analysis.

### 3.3. Preparation of Solutions

Krebs–Henseleit buffer (KHB) of pH 7.4 was prepared with the following composition: 118.1 mM NaCl, 4.69 mM KCl, 1.2 mM KH_2_PO_4_, 25.0 mM NaHCO_3_, 11.7 mM glucose, 1.2 mM MgSO_4_, and 2.5 mM CaCl_2_. DAF-FM was prepared as a stock solution (5 mM) in DMSO, divided into aliquots and stored at −20 °C, followed by dilution to the required concentration in buffer before use. A 0.02% (*w*/*v*) solution of Pluronic F-127^®^ 20% (*w*/*v*) solution in DMSO was added to the final DAF-FM solution. Pluronic F-127^®^ is a nonionic surfactant which helps to increase the solubilization of water-insoluble dyes and other materials in physiological media. SIN-1 was prepared as stock solution (10 mM) in DMSO, divided into aliquots, and stored at −80 °C prior to dilution. Ten microliters of SIN-1 stock solutions were mixed with 10 mL saline or KHB, respectively, to prepare the working solutions (each at 1 µM). ACh was prepared as a stock solution (30 mM) in deionized water, divided into aliquots, and stored at −20 °C prior to dilution to the final concentration before use with KHB containing DAF-FM.

### 3.4. Nitric Oxide Generation

This study used SIN-1 in solution to liberate NO to optimize the condition for DAF-FM/NO reaction. The reaction factors studied include the optimum excitation and emission wavelengths, the effect of dye concentration, and buffer type. Having established the factors that optimized the method, they were used to measure NO generated from aorta that had been physically stimulated by ACh.

#### 3.4.1. Optimization of Excitation and Emission Wavelengths

The optimum excitation and emission wavelengths were determined by measuring the fluorescence intensity of the reaction product of 2.5 µM DAF-FM with SIN-1-derived NO at different excitation (480–500 nm) and emission wavelengths (515–555 nm).

#### 3.4.2. Effect of Dye Concentration

The first concentration (10 µM) of DAF-FM working solution was prepared by mixing 2 µL of the 5.0 mM DAF-FM stock solution with 1 mL of KHB containing 0.01% Pluronic F-127^®^ under low ambient light conditions. Serial dilutions of the DAF-FM working solution (to final concentrations of 0.31–5 µM) were prepared by mixing 500 µL of the working solution (10 µM) with appropriate volumes of KHB containing Pluronic F-127^®^. Ninety microliters of each of the prepared DAF-FM solutions were transferred to 96-well black plates in triplicates. Ten microliter aliquots of the prepared solutions of SIN-1 (each at 1 µM) were transferred to the 96-well plates containing the different concentrations of DAF-FM. After 10 s of plate shaking, the fluorescence intensity was measured at λ_ex_ = 495 nm and λ_em_ = 525 nm.

#### 3.4.3. Effect of Buffer

Different buffer systems (KHB, PBS, and saline) were used for the preparation of DAF-FM working solutions, as described above. After 10 s of plate shaking, the fluorescence intensity was then measured at λ_ex_ = 495 nm and λ_em_ = 525 nm.

#### 3.4.4. Effect of Reaction Time and Fluorescence Stability

The time required to complete the reaction between DAF-FM and the NO in KHB was determined by continuously measuring the fluorescence intensity over a 60 min period. The fluorescence stability of the obtained product was investigated by measuring the fluorescence intensity at the end of this time using different numbers of flashes [1,2,3,4,5,6,7,8,9].

### 3.5. Validation Study

The developed analytical method was validated by means of linearity, specificity, precision, limit of detection (LOD) and limit of quantification (LOQ), as described in the International Conference on Harmonisation (ICH) guidelines. In order to study the linearity, the fluorescence intensity was measured as a function of concentration of the NO liberated from the NO donor, which reacted with DAF-FM under the optimized conditions. The linearity of the method was studied at the NO range 1–10 nM, as it is the common range of NO released from vascular endothelium. Statistical parameters and linearity results of the standard curve (1–10 nM) were determined. Relative fluorescence intensity is the fluorescence measured at emission wavelength 525 nm with excitation at 495 nm after subtracting blank values.

### 3.6. Application of the Optimized Method in Measuring the Generation of Vascular Endothelial NO

Animal study: The study is reported in accordance with the Kingdom of Saudi Arabia Research Bioethics and Regulations. Male Wistar rats (6 weeks of age; King Abdulaziz University, Jeddah, Saudi Arabia) were housed (three to four rats per cage) in clear polypropylene cages and kept under constant environmental conditions with equal light–dark cycle. Rats had free access to commercially available rodent pellet diet and purified water. Rats were killed by decapitation with rodent guillotine, and the descending thoracic aorta was carefully excised and placed in cold KHB. The aorta was then cleaned from fat and connective tissue, then cut into rings (~3 mm length).

NO generation: The optimized method for measuring NO liberated from SIN-1 as developed and described herein was subsequently applied to endothelial NO generated from the vascular endothelium. To this end, 300 µL of 2.5 µM DAF-FM in KHB was added to a set of wells in a black 96-well plate. One isolated aortic ring was inserted in each well for 3 min, then 100 µL of the medium was transferred to new wells for spontaneous fluorescence intensity measurements. Then, 100 µL of ACh (30 µM) prepared in KHB/DAF-FM was added to each well containing the isolated aortic ring (10 µM ACh final concentration), followed by transferring 100 µL of the medium to new wells and the measurement of fluorescence intensity. This was repeated with ACh concentrations 300 and 3000 µM to give final concentrations of 100 and 1000 µM.

## 4. Conclusions

Because of the role of NO in vascular health, it is important to be able to quickly, accurately, and reliably quantify the levels of NO generated by vascular endothelial tissue. Our optimized method presented here provides a high-throughput means to measure NO using the microplate reader, which is valuable because it allows a large number of samples to be run simultaneously. It also allows the analyst to use microliter volumes of samples, so it is considered to be an easy, reliable, time saving method for NO measurement. In addition, this method can be used to endothelial NO generation under the effect of different inhibitors or injury substances.

## Figures and Tables

**Table 1:** Statistical parameters and linearity results based on the standard curves of the fluorescence intensity of the reaction between DAF-FM and the NO liberated from SIN-1 (over the range 1–10 nM). LOD: limit of detection; LOQ: limit of quantification.

Parameter		SIN-1 (nM)
Slope		4.862 ± 0.3808
*y*-intercept		8.432 ± 2.311
*x*-intercept		−1.734
Coefficient of determination	r^2^	0.9761
	*p*	0.0002
LOD (nM)		0.20
LOQ (nM)		0.61
Precision % RSD (intra-day)		2.45
% RSD (interday)		3.56
